# Lipids Nutrients in Parkinson and Alzheimer’s Diseases: Cell Death and Cytoprotection

**DOI:** 10.3390/ijms21072501

**Published:** 2020-04-03

**Authors:** Thomas Nury, Gérard Lizard, Anne Vejux

**Affiliations:** Biochemistry of the Peroxisome, Inflammation and Lipid Metabolism’ EA 7270, Team Bio-peroxIL, Université de Bourgogne Franche-Comté /Inserm, 21000 Dijon, France; thomas.nury@u-bourgogne.fr

**Keywords:** lipids nutrients, apoptosis, oxidative stress, mitochondria, synuclein, amyloid, Tau, Parkinson’s disease, Alzheimer’s disease

## Abstract

Neurodegenerative diseases, particularly Parkinson’s and Alzheimer’s, have common features: protein accumulation, cell death with mitochondrial involvement and oxidative stress. Patients are treated to cure the symptoms, but the treatments do not target the causes; so, the disease is not stopped. It is interesting to look at the side of nutrition which could help prevent the first signs of the disease or slow its progression in addition to existing therapeutic strategies. Lipids, whether in the form of vegetable or animal oils or in the form of fatty acids, could be incorporated into diets with the aim of preventing neurodegenerative diseases. These different lipids can inhibit the cytotoxicity induced during the pathology, whether at the level of mitochondria, oxidative stress or apoptosis and inflammation. The conclusions of the various studies cited are oriented towards the preventive use of oils or fatty acids. The future of these lipids that can be used in therapy/prevention will undoubtedly involve a better delivery to the body and to the brain by utilizing lipid encapsulation.

## 1. Introduction

Neurodegenerative diseases, a major societal issue, are pathological conditions that affect neurons, condemning them to certain death. This term covers a heterogeneous group of diseases affecting different populations of neurons of the nervous system (brain and spinal cord), i.e., around 100 pathologies. In this review, we will focus on the most frequent: Alzheimer’s disease and Parkinson’s disease. The mechanisms of cell death have been well-studied in vitro and in vivo, both in animal models of these pathologies and in humans. Many mechanisms are common to these different pathologies; what differentiates them is the type of neurons affected and its environment. Parkinson’s disease is a chronic pathology characterized by the destruction of dopaminergic neurons at the level of the substantia nigra. These innervate the central grey nuclei and, more particularly, a structure involved in the control of motor functions, the striatum. One of the common mechanisms of neurodegeneration is the toxic aggregation of proteins. Once their synthesis is complete, proteins acquire a three-dimensional organization that allows them to perform their function. Under normal conditions, if misfolding occurs, it is either corrected or the misfolded proteins are eliminated by either the ubiquitin-proteasome pathway or by autophagy. Under pathological conditions, these misfolded proteins are not eliminated; they will aggregate and take the form of intranuclear or intracytoplasmic inclusions, or extracellular aggregates called senile plaques, which inhibit many subcellular functions [1,2,3]. For example, in Alzheimer’s disease, there are senile plaques and tau protein aggregates. In Parkinson’s disease, there are α-synuclein aggregates in the form of Lewy bodies or Lewy neurites. A loss of activity of these proteins, a gain in neurotoxic function or the induction of an inflammatory process could explain the toxicity of these aggregates. These aggregates could also induce apoptosis of neurons associated with mitochondrial dysfunction; another process common to various neurodegenerative pathologies. Since the mitochondria are closely related to the peroxisomes, mitochondrial dysfunction can adversely affect peroxisomal activity. The third common process in neurodegeneration is the presence of oxidative stress with increased production of reactive oxygen species: the superoxide ion O_2_^.-^, the peroxynitrite ion ONOO-, the hydroxyl radical HO and other molecular species such as hydrogen peroxide H_2_O_2_. Firstly, this review will present the commonalities between Parkinson’s and Alzheimer’s disease: protein accumulation, mitochondrial dysfunction and cell death, followed by oxidative stress. Subsequently, the lipids that can protect against cell death will be discussed in the form of vegetable, animal and functionalized oils and by individual fatty acids.

## 2. Mechanisms Common to Neurodegenerative Diseases

Among the mechanisms common to the two most-frequent neurodegenerative diseases in humans, Parkinson’s and Alzheimer’s disease, protein aggregation associated with altered protein degradation systems, mitochondrial dysfunction associated with cell death and oxidative stress were identified. These characteristics and common features will be described for these two neurodegenerative pathologies.

### 2.1. Protein Aggregation and Alteration of Protein Degradation Systems

In Parkinson’s and Alzheimer’s disease, the proteins that primarily aggregate are α-synuclein, Tau and β-amyloid proteins, respectively, although the latter are also found in Parkinson’s disease. Insoluble α-synuclein fibrils make up the Lewy bodies and Lewy neurites. These are predominantly present in the pigmented neurons of the substantia nigra and in other neuronal populations at the peripheral and central levels [4,5,6]. Lewy bodies are present in the dopaminergic neurons of substantia nigra pars compacta and are round intraneuronal and positive round inclusions of α-synuclein and ubiquitin [6]. Lewy neurites are abnormal neurites with α-synuclein filaments and granular material that accumulate in the amygdala and striatum of Parkinsonian patients [7]. Lewy neurites can inhibit neuronal transport and, thus, compromise neuronal function and survival. In Alzheimer’s disease, the alterations identified are amyloid plaques and neurofibrillary degeneration. The role of two proteins have been identified: Tau protein and Amyloid precursor protein (APP). The APP protein can be proteolyzed to generate the Aβ peptide. When this peptide aggregates, amyloid plaques are formed [8]. Neurofibrillary degeneration consists of argentophilic neurofilaments located in the pericaryon of certain cortical neurons but are also found within myelinated axons, dendrites and synapses [9]. The density of these neurofibrillary accumulations in the neocortex is directly correlated with the severity of the disease [10]. Neurofibrillary tangles consist of hyper-phosphorylated Tau proteins organized in paired helical filaments. Amyloid plaques consist of extracellular deposits of peptide Aβ. These plaques are surrounded by glial cells containing phagocytic lysosomes, as well as amyelinic neurites. These plaques are preferentially located in certain areas of the brain, particularly the cortex, striatum and cerebellum. They undergo maturation during their evolution and, thus, present a specific morphology. The less mature plaques are referred to as diffuse plaques. They are formed of amorphous, low-density aggregates of peptides Aβ. Mature plaques contain very dense aggregates of the peptide Aβ. They are associated with neurodegeneration and astroglial reactivity.

There are different systems for the removal of malformed proteins, including the two pathways involved in the degradation of α-synuclein: the ubiquitin-proteasome system (UPS) and the autophagy-lysosome pathway (ALP) [11,12]. What differs between these two pathways is the type of proteins that are recognized—for the UPS system: short-lived soluble proteins and for the ALP pathway: long-lived macromolecules, cytosolic components and dysfunctional organelles. In the case of Parkinson’s disease, one of the pathways of the ALP system, chaperone-mediated autophagy (CMA), is involved. It is known that CMA activity is linked to levels of LAMP-2A (the receptor of the CMA pathway which bind to chaperones who have recognized proteins possessing the KFERQ motif), and in the substantia nigra of patients’ brains, the expression of LAMP-2A is particularly diminished [13,14,15]. This reduction is thought to be related to the accumulation of α-synuclein and nigral cell death [16]. In addition to the deregulation of the CMA system, an accumulation of autophagosomes and changes in enzyme content and acidification of the lysosomes were observed [17,18,19]. In Alzheimer’s disease, it has been suggested that proteasome inhibition may explain the accumulation of Aβ inside cells in multivesicular bodies [20]. Similarly, proteasome inhibition, both in vitro and in vivo, increases peptide levels Aβ [21]. In addition, extracellular Aβ entering the cytosolic compartment can inhibit proteasome activity in cultured neurons, leading to its accumulation in the cytosol [21].

### 2.2. Mitochondrial Dysfunction and Cell Death

In the case of Parkinson’s disease, it has been established that mitochondria are involved via a deficiency in the complex I of the respiratory chain at the level of the patient’s black matter in the neurons and in the glia but, also, at the level of skeletal muscle and platelets [22,23,24,25]. This dysfunction contributes to the increase of mitochondria-dependent apoptotic processes [26]. Changes in mitochondrial dynamics, at the bioenergetic level, as well as the inhibition of complex I of the respiratory chain, have been identified on experimental models but, also, in patients [27,28]. Moreover, the genes involved in Parkinson’s disease are important for mitochondrial function. For example, mutations in the LRRK2 gene are associated with alterations in the mitochondria [29]. The proteins encoded by the PARK2 and PINK1 genes are involved in the clearance of mitochondria damaged by mitophagy [29]. In patients with Parkinson’s disease, degradation of the MIRO protein (outer mitochondrial membrane protein involved in the binding between the mitochondria and microtubules) is reduced, leading to loss of mitophagic function, which may result in increased oxidative stress [30]. Beyond the involvement of genetic factors and observed functional changes, α-synuclein can also interact with the mitochondria and modulate their function. α-synuclein can be located at the mitochondrial outer membrane, interact with members of the TOM complex (translocase/receptor system) and, thus, inhibit the mitochondrial import of proteins in Parkinson’s disease [31]. These interactions between mitochondria and α-synuclein may also be accompanied by excessive production of oxidative stress [32]. α-synuclein can also disrupt mitochondrial dynamics, particularly the fusion process, and inhibits complex I [33,34]. Histological evidence implicates cell death as the process responsible for Parkinson’s disease, as indicated by the presence of fragmented DNA and, therefore, apoptosis in patients’ brains (TUNEL methods) and of active forms of caspases -1, -3, -8 and -9 at the level of the black substance [35,36]. It was also shown that the mitochondrial pathway (p53-GAPDH-Bax pathway) is involved, as is the Fas/FADD death receptor pathway, again thanks to post-mortem studies (high levels of Fas, FADD and caspase-8 in the brains of Parkinsonian patients) [37].

In Alzheimer’s disease, a major feature is a loss of neurons in the cortical II layer of the entorhinal cortex [38]. Neurons in the entorhinal cortex that synthesize acetylcholine and innervate the hippocampus and neocortex can also die in Alzheimer’s disease. The accumulation of amyloid plaques and neurofibrillary degeneration would be the main trigger for neuronal death of these cells. Neuronal death can be induced by exogenous Aβ and pseudo-hyperphosphorylated Tau, as shown in various cell cultures and animal models of Alzheimer’s disease [39]. Indeed, neurons can internalize the extracellular Aβ peptide [40]. The Aβ peptide could thus disrupt mitochondrial membranes and alter the enzymatic activities of the respiratory chain associated with it, thereby leading to the production of a reactive oxygen species [41]. In response to induced oxidative stress, various enzymes, including calpains and caspases, are activated, leading to the cell death of neurons [42].

### 2.3. Oxidative Stress

Oxidative stress is a process induced by different free radicals, reactive oxygen species (ROS) and reactive oxygen and nitrogen species (RONS). Among these species are hydrogen peroxide (H_2_O_2_), which, in the presence of iron (in ionic form), gives two hydroxyl radicals (^.^OH), nitrogen monoxide (NO^.^) and superoxide (O_2_^.−^). Free radicals cause damage to proteins, lipids and DNA. This type of damage is found in the substantia nigra of Parkinson’s patients. This would contribute to neuronal degeneration. At the level of patients’ substantia nigra, damage to proteins, lipids and DNA related to free radicals has been identified in postmortem studies [43]. The dysfunctions of the complex I of the mitochondria mentioned earlier induce an increase in the production of free radicals, and conversely, oxidative stress leads to dysfunction of the mitochondria [43]. As in the case of mitochondria, genes may be involved in setting up oxidative stress. Mutations in the DJ-1 or PARK7 genes, coding for a protein suspected of having antioxidant activity, are concomitant with increased oxidative stress. In DJ-1-deficient mice, there is increased protein oxidation in stressed dopaminergic neurons [44]. Mitochondrial dysfunction and oxidative stress can lead to lysosome depletion and functional impairment of the autophagy/lysosome system, highlighting the interconnection between the different processes responsible for the pathology [45].

In Alzheimer’s disease, in addition to the activation of certain enzymes indicated in the previous paragraph, ROS disrupts the cytoskeleton, which undergoes various disorganization phenomena, particularly at the level of dendrites [42] but, also, the plasma membrane and the membranes of the cell organelles. This activates the BACE protein and, consequently, diverts the metabolism of the APP protein towards the amyloidogenesis pathway with increased production of the peptide Aβ. As in the case of Parkinson’s disease, oxidative damage has been found in proteins, nucleic acids and lipids [46,47]. The Aβ peptide can also act as a pro-oxidant directly or by impacting NMDA receptor-dependent calcium influxes, leading to mitochondrial dysfunction and, thus, ROS production [48,49].

### 2.4. Inflammation and Immunity

The central nervous system (CNS) has an endogenous immune system coordinated by immunocompetent cells. The main actors involved in the inflammatory process during Alzheimer’s disease are microglial cells, glial cells (astrocytes) and, possibly, neurons [50,51]. Both microglial cells and astrocytes respond rapidly to the disease with changes in morphology, antigenicity and function [52]. Microglial cells are cells that support and protect neurons. They act as immunocompetent defense cells that orchestrate the endogenous immune response of the CNS. They can express major histocompatibility complex type II (MHCII) and produce proinflammatory cytokines, chemokines, ROS and complement proteins. These cells play a central role in the cellular response to pathological lesions such as Aβ and senile plaques [53]. In fact, Aβ can attract and activate microglial cells, leading to their accumulation around amyloid deposits. Exposure of microglial cells to Aβ causes their activation, leading to increased MHC II cell expression and increased secretion of proinflammatory cytokines (interleukin-1 (IL-1), interleukin-6 (IL-6) and TNF-α (tumor necrosis factor-α)), as well as chemokines (interleukin-8 (IL-8), MIP-1α (macrophage inflammatory protein-1α) and MCP1 (monocyte chemoattractant protein-1)) [54,55]. Aβ may promote passage of peripheral circulating macrophages through the blood-brain barrier following recruitment by chemokines, which could increase the extent of inflammation. Kopec & Carroll (1998) showed that Aβ can induce a phagocytic response in microglial cells in a dose- and time-dependent manner [56]. Internalization and co-localization of the lysosome and Aβ associated with damage to neighboring neurons were observed by Bolmont et al., which coincided with the arrival of microglial cells, while plaque resolution was not observed for weeks [57,58]. Reactive astrocytes also contribute to neurodegeneration by stimulating apoptotic processes [59]. In Alzheimer’s disease, astrocytes are found associated with amyloid deposits, where they secrete several proinflammatory molecules such as interleukins, prostaglandins, leukotrienes, thromboxanes and complement factors. These molecules are like, and co-localized with, those secreted by microglial cells. One study detected diffuse plaques associated with astrocytes, consisting of granules of Aβ, in the brains of nondemented elderly people. This observation suggests that astrocytes may be involved in the phagocytosis of Aβ [60] and that, probably, a deficit in the elimination of Aβ by astrocytes is part of the pathology of Alzheimer’s disease. In the brains of Alzheimer’s disease patients [52] and transgenic models of Alzheimer’s disease mice [61], reactive astrocytes occupy positions near the plaques, encircling the deposits of Aβ, a mechanism by which the cells could act as a barrier between healthy tissue and tissue with inflammation [52]. In addition, the fibrillar form of amyloid peptide interacts with complement proteins and thereby activates the innate immune system [62]. In particular, the interaction Aβ/C1q, which involves the 11 N-terminal residues of Aβ, activates the classical complement pathway [63]. This process induces a cascade of activations that leads to the formation of lytic complexes, partly responsible for neuronal death, and inflammatory activation [64]. Neurons themselves may play a role in the inflammatory process through the production of certain cytokines such as IL-1 [65], IL-6 [66], TNF-α [67] and some pentraxins, namely CRP and Ap (amyloid P) [68]. The mRNA expression levels of proteins of the classical complement pathway are similarly increased in the brains of Alzheimer’s patients compared to those of controls [69].

Numerous studies have implicated neuroinflammation and, more specifically, microglial cells in the development and progression of death of the dopaminergic neurons of the substantia nigra [70]. Positron emission tomography (PET) imaging has made it possible to visualize microglial activation in the pons, central gray nuclei, striatum and frontal and temporal cortical regions of patients with Parkinson’s disease [71,72]. Post-mortem immunohistological analyses also show morphological and functional signs of microglial activation in the CNS: increased cell density, retraction of branches and hypertrophy of the cell body; expression of MHC II molecules [73,74,75,76] and increased expression of the lysosomal marker CD68 [75,77]. Phagocytosis and antigenic presentation are classically the two main functions attributed to microglial cells. In the 6-hydroxydopamine (6-OHDA) model, analysis of the lysosomal marker CD68, reflecting phagocytosis activity, and of MHC II molecules, reflecting the role of antigen presentation, shows that these two functions may evolve according to independent kinetics [78,79,80,81]. Peak phagocytic activity, as determined by the level of CD68 expression, has been reported to occur either before or after maximal neuronal death [79,82]. These contradictory results lead to conflicting hypotheses as to the role of microglial activation. It might be assumed that a process of deregulation of phagocytic functions amplifies the autonomous mechanisms of neuronal death. It could also be assumed that the main function of microglial cells is the removal of neuronal cell debris. Activated microglia also have the important property of secreting a set of cytokines that are either proinflammatory or anti-inflammatory. Potentially neurotoxic proinflammatory proteins are expressed in the brains of patients with Parkinson’s disease, including COX and iNOS, TNFα, IL1β and IFNγ [83,84]. T-lymphocytes are the main cells that produce IFNγ, a cytokine known to activate macrophages (including microglia). Numerous animal studies have demonstrated a deleterious role of IFNγ in the pathophysiology of Parkinson’s disease [85,86,87,88]. Since Th1 lymphocytes are the main source of IFNγ and macrophages are the main target cell for this cytokine, these results suggest that the dialogue between Th1 and microglial cells in the substantia nigra of patients with Parkinson’s disease could be a key event in dopaminergic neuronal loss. In the MPTP model, the regulatory T-lymphocytes (Tregs) prevent the degeneration of dopaminergic neurons by altering the molecular behavior of microglial cells [89,90]. Thus, microglial cells are likely to exert distinct and possibly opposing effects depending on the stage of Parkinson’s disease progression, the immunogenetic terrain and a set of instructional signals delivered by infiltrating T lymphocytes, among other factors.

## 3. Cytoprotector Effects of Vegetable Oil

**Figure 1:**
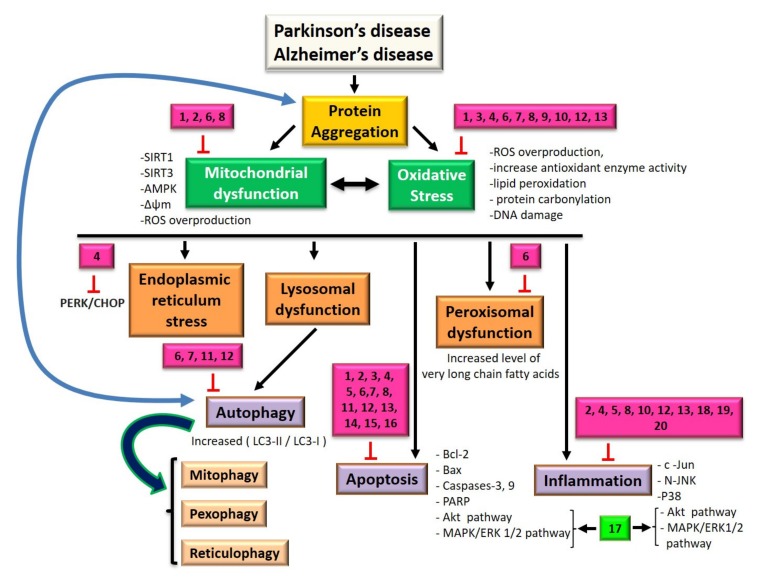
Signaling pathways impacted by lipid nutrients at the level of target organelles and common processes involved in Parkinson’s and Alzheimer’s disease: autophagy, apoptosis, oxidative stress and inflammation. (**1**) Compounds derived from sesame oil and sesame seeds, (**2**) red ginseng oil, (**3**) coriander volatile oil, (**4**) compounds of *Nigella sativa* seed oil, (**5**) olive oil, (**6**) argan oil, (**7**) milk thistle seed oil, (**8**) krill oil, (**9**) sea urchin egg oil, (**10**) compounds of Lorenzo’s oil, (**11**) oleic acid, (**12**) docosahexaenoic acid, (**13**) eicosapentaenoic acid, (**14**) α-linolenic acid, (**15**) linoleic acid, (**16**) arachidonic acid, (**17**) plasmalogens, (**18**) compounds of *Cyperus rotundus* oil, (**19**) compounds of dietary tuna oil and (**20**) fish oil.

In this paragraph, we will discuss the effects of oils or molecules derived from vegetable oils as a potential therapeutic or preventive avenue against cell death (mitochondria, apoptosis and oxidative stress) and inflammation. These effects are reported in Figure 1, which summarizes the different signaling pathways potentially involved in Parkinson’s and Alzheimer’s disease, as well as the sites of action of lipid nutrients that will be described in this section (Figure 1).

In SH-SY5Y cells, sesamin and sesamol (concentration 1 µM), two compounds derived from sesame oil and sesame seeds, are capable of reducing H_2_O_2_-induced cell death, as well as the production of intracellular ROS [91]. The signaling pathway involved in this neuroprotection involves activation of SIRT1-SIRT3-FOXO3a expression, inhibition of the proapoptotic protein Bax and positive regulation of the antiapoptotic protein Bcl-2 [91]. In 6-OHDA-treated rats (PD model), sesamin decreases ROS levels, increases superoxide dismutase activity and decreases caspase-3 activity [92]. In another cellular model of Parkinson’s disease (use of 1-methyl-4-phenyl-pyridine (MPP(+)) ion), sesamin also protects PC12 cells from cell death by reducing oxidative stress [93].

Red ginseng oil is able to protect PC-12 cells treated with Aβ(25–35) peptides from cell death. This oil attenuates apoptosis by acting on calcium influx and loss of mitochondrial membrane potential but, also, on the proteins involved in the death pathways: Bax, Bcl-2 and caspases-3 and -9, as well as PARP [94]. It also has an anti-inflammatory effect by negatively regulating the c-Jun/N-JNK/p38 pathway [94]. Red ginseng oil extract is composed mainly of linoleic acid, β-sitosterol and stigmasterol. These three compounds inhibit the toxicity induced by Aβ(25–35) by regulating oxidative stress, apoptosis and inflammation but using different molecular mechanisms in PC12 cells [95]. For example, at the level of cell death, linoleic acid and stigmasterol regulate mitochondrial membrane potential; intracellular calcium; Bax/Bcl-2 ratio and caspases-9, -3 and -8, while β-sitosterol blocks only the intrinsic apoptotic pathway [95].

Inhaled coriander volatile oil was tested in a rat model for Alzheimer’s disease and decreased markers of oxidative stress on hippocampal tissues: decreased superoxide dismutase and lactate dehydrogenase activities, increased glutathione peroxidase activity and decreased malondialdehyde levels [96]. This inhaled oil may also decrease antiapoptotic activity [96]. Studies on *Coriandrum sativum* leaves have confirmed that this species has antioxidant, anti-inflammatory and, especially, ERK-signaling inhibitory properties that are beneficial for Alzheimer’s patients [97].

Thymoquinone is the most abundant ingredient in *Nigella sativa* seed oil. This molecule protects human-induced pluripotent stem cell (hiPSC)-derived cholinergic neurons treated with Aβ(1–42) from cell death, oxidative stress and synaptic toxicity [98]. Thymoquinone reduces inflammation in a model of Alzheimer’s disease by likely acting on the TLR pathway and NF-κB-signaling pathway [99,100].

Total ginsenosides and volatile oil of *Acorus tatarinowii* administered together to AD mice inhibit apoptosis, decrease malondialdehyde content in the cortex and hippocampus and increase superoxide dismutase activity [101]. *Acorus tatarinowii* Schott volatile oil, via its compound β-asarone (1,2,4-trimethoxy-5-([Z]-prop-1-enyl)benzene), is able to regulate autophagy and stress of the endoplasmic reticulum in a 6-OHDA-induced Parkinsonian rat model by inhibiting the PERK/CHOP/Bcl-2/Beclin-1-signaling pathway [102].

Olive oil, a major constituent of the Mediterranean diet, through its two components tyrosol and hydroxytyrosol, decreases the cell death of N2a cells treated with Aβ(25–35) but cannot prevent the decrease of glutathione induced by H_2_O_2_ or Aβ [103]. In SH-SY5Y cells treated with dopamine and 6-hydroxydopamine, hydroxythyrosol protects against death induced by these compounds [104]. Oleocanthal, one of the active components of extra-virgin olive oil, reduces interleukin-6 increase and glial fibrillary acidic protein upregulation, characteristic processes of inflammation induced by Aβ oligomers [105].

α-cyperone, one of the main ingredients of *Cyperus rotundus* oil, exerts neuroprotective effects in BV-2 microglial cells by reducing the production of inflammatory cytokines: IL-1β, TNF-α and IL-6. This action occurs by activating Akt/Nrf2/HO-1 and suppressing the NF-κB pathway [106].

The consumption of dietary tuna oil (0.55% EPA and 0.36% DHA, % of total diet weight) for eight weeks, in a mouse model of age-related pathologies such as Alzheimer’s disease, prevents elevation of hippocampal TNF-α and monocytic marker CD11b protein levels, whereas GFAP and IL-1β increase despite this dietary supplementation [107].

Some oils have not been tested in a model for Parkinson’s or Alzheimer’s disease, but their actions have been evaluated in cell models where oxidative stress and organelle dysfunction have been induced by oxysterols (oxidized derivatives of oxysterols) in cells of the nervous system: oligodendrocytes. Indeed, when their concentration is abnormal, oxysterols are associated with demyelinating or neurodegenerative diseases such as Parkinson’s or Alzheimer’s disease [108,109]. 7-ketocholesterol (7KC) is capable of inducing cell death and oxidative stress in 158N oligodendrocyte cells [110].

Argan oil is capable of inhibiting processes induced by 7KC: loss of cell adhesion; cell growth inhibition; increased plasma membrane permeability; mitochondrial, peroxisomal and lysosomal dysfunction and the induction of oxiapoptophagy (OXIdation + APOPTOsis + autoPHAGY) in oligodendrocytes [111].

In the 158N oligodendrocyte cell model, OSS of cell adhesion, increased plasma membrane permeability, mitochondrial dysfunction, ROS overproduction, induction of apoptosis and autophagy induced by 7KC are attenuated by milk thistle seed oil [112].

All these oils can be potential natural resources for the functionalized food industry, as well as being used as neural protective agents.

## 4. Cytoprotective Effects of Animal Oils

Seafood can have neuroprotective effects. Data are available, not on models strictly mimicking Alzheimer’s and Parkinson’s disease, but on models adapted to resemble certain features of neurodegenerative diseases. These effects are reported in Figure 1.

Krill oil protects PC-12 cells from methamphetamine-induced cell death by increasing cell viability, decreasing cleaved caspase-3 and regulating mitochondrial membrane potential [113]. It also protects from oxidative stress by increasing superoxide dismutase and glutathione enzyme activities and decreasing ROS and NO production [113]. The authors suggest that apoptosis and oxidative stress are key processes in the pathophysiology of many neurodegenerative diseases and that this oil could be beneficial in these pathologies [113]. Antarctic krill oil reduces oxidative stress in the brains of SAMP8 mice, but the effects are barely visible in the serum and liver [114].

Fish oil decreases the expression of neuroinflammatory genes in response to amyloid-β [115]. Short-term fish oil supplementation, used in the presymptomatic stage of Alzheimer’s disease, influences the behavior of microglia/macrophages, inducing them to establish a physical barrier around the amyloid plaques [116]. In the mu-p75 saporin (SAP)-induced mouse model of Alzheimer’s disease, a mixture of tart cherry extract, Nordic fish oil and refined emu oil protects from inflammation and the loss of neurons induced by SAP [117]. Sprague Dawley rats, aged eight weeks and LPS-injected, were fed for two weeks with food containing 15% fish oil (% of total diet weight). Dopaminergic lesions were decreased in this model. as well as OX42 (also known as CD11b) protein, a monocytic marker, TNF-α and IL-1β [118].

Oxysterols may be involved in the development of neurodegenerative diseases [108]. Based on this hypothesis, mouse 158N oligodendrocytes were treated with 7β-hydroxycholesterol. This induces cell death associated with oxidative stress (alterations in superoxide dismutase and glutathione peroxidase activities, increased lipid peroxidation and protein carbonylation) [119]. Sea urchin egg oil attenuates the cytotoxicity induced by 7β-hydroxycholesterol. This result led to the hypothesis that this oil could be useful in the prevention of neurodegenerative diseases and, therefore, possibly Alzheimer’s and Parkinson’s disease [119].

## 5. Cytoprotective Effects of Functionalized Oil

Functionalized oils, or compounds of these oils, may eventually become therapeutic avenues through their actions on cell death and oxidative stress. These actions are reported in Figure 1.

Erucic acid (monounsaturated ω9-fatty acid, denoted 22:1ω9) is one of the components of Lorenzo’s oil, used in adrenoleukodystrophy. It is consumed in some Asian countries and by the Eskimos of Greenland as an edible oil. Erucic acid has an antioxidative activity that could counteract the oxidative stress present in neurodegenerative diseases, particularly Alzheimer’s disease [120]. Indeed, it has been shown in various studies that erucic acid could reduce oxidative damage to DNA and is capable of modifying catalase activity (a peroxisomal enzyme involved in the protection of cells against ROS) but also of protecting the mitochondrion and stimulating its biogenesis (via its PPARδ ligand action) [120]. Erucic acid, present at high levels in mustard, can also inhibit inflammatory enzymes. Many oils contain erucic acid, such as corn oil, rapeseed oil, soybean oil, safflower oil, perilla oil and mustard oil, and it has been shown that these oils can have a positive effect on locomotor activity and memory [120].

## 6. Cytoprotective Effects of Fatty Acids

In the case of individual lipids, which are components of oils or seafood products, various studies have been carried out on cells of the nervous system, suggesting possible actions in Parkinson’s and Alzheimer’s disease. Their actions are reported in Figure 1.

In a mouse microglial cell model BV-2, oleic acid (OA), a major compound of olive and argan oil, and docosahexaenoic acid (DHA; C22:6 n-3) present in fatty fishes, such as sardines, are able to inhibit the major toxic effects of 7KC: caspase-3 activation and increased LC3-II/LC3-I ratio (autophagy indicator) [121].

Some n-3 polyunsaturated fatty acids (PUFAs), such as docosahexaenoic acid (DHA) and eicosapentaenoic acid (EPA; C20:5 n-3), are known to have beneficial effects in neurodegenerative diseases. In a model using differentiated SH-SY5Y cells treated with Aβ(25–35), DHA, EPA and mixtures of these two compounds at DHA/EPA ratios 1:1 and 2:1 are able to modulate the neurotoxicity of the Aβ(25–35) peptide by decreasing cell death (Bax/Bcl-2 ratio and caspase-3) and oxidative stress [122]. DHA and EPA can be provided in different forms (glycerophosphatides, triglycerides or ethyl esters). The effects of these different modifications are considered. Chinese hamster ovary cells (CHO) stably transfected with amyloid precursor protein (APP) and presenilin 1 (PS1) and SAMP8 mice fed with high-fat diets (HFDs, known to induce metabolic stress, leading to cognitive impairment and aging) have been used to mimic Alzheimer’s disease. DHA-enriched phosphatidylcholine (DHA-PC) and EPA-enriched phosphatidylcholine (EPA-PC) were tested in these models, and it was shown that these two compounds can reduce oxidative stress by inducing antioxidant systems (SOD, T-AOC, GSH and GSH-PX) and inhibiting oxidative systems (reduced MDA, NO and NOS levels). They also inhibit apoptosis [123]. In an Alzheimer’s rat model (induction Aβ1–42), EPA-PC and DHA-ethyl ester are able to decrease lipid peroxidation rates and mitochondrial-dependent cell deaths [124]. In HFD-induced Alzheimer’s disease mice models, DHA-PC and DHA-phosphatidylserine (DHA-PS) can inhibit oxidative stress, and DHA-PS is more effective than DHA-PC in the inhibition of mitochondrial damage [125].

One study tested the abilities of PUFAs n-3 and n-6: DHA, EPA, α-linolenic acid (α-LNA; C18:3 n-3), linoleic acid (LA; C18:2 n-6), arachidonic acid (AA; C20:4 n-3) and γ-linolenic acid (γ-LNA; C18:3n-6) to inhibit cell death. Different known inducers of cell death etoposide, okadaic acid and AraC have been used in mouse neuroblastoma cells (Neuro2a). All these PUFAs inhibit cell death when used separately but had no effect when used together. These results therefore prompted the authors to propose PUFAs as molecules that could delay the onset of diseases and/or their rates of progression [126].

The use of DHA and EPA, for six months, prevents a decrease in the levels of specialized protective mediators, involved in inflammation, produced by peripheral blood mononuclear cells (PBMC) of Alzheimer’s patients [127]. This effect is accompanied by improvements in cognitive function [127]. EPA supplementation appears to improve glial overactivation and to have effects on n3/n6 imbalance and the negative regulation of BDNF, which contribute to the anti-inflammatory actions [128]. The same supplementation, given as part of the OmegAD study, induces the regulation of different genes involved in inflammation, e.g., CD63, MAN2A1, CASP4, LOC399491, NAIP and SORL1 and in ubiqutination processes, e.g., ANAPC5 and UBE2V1 [129]. Additionally, DHA and EPA supplementation decreased the release of IL-1β, IL-6 and granulocyte colony-stimulating factor from the PBMC of Alzheimer’s patients [130]. DHA, in combination with (-)epigallocatechin-3-gallate and α-lipoic acid promoted anti-inflammatory (microglial activation) and neuroprotective effects in male Tg2576 transgenic mice [131]. Human CHME3 microglial cells, treated with the peptide Aβ42, also received DHA and EPA. This combination of omega-3 fatty acids stimulates microglial phagocytosis of Aβ42, decreases proinflammatory M1 markers CD40 and CD86 and increases neurotrophin production [132].

A model of Parkinson’s disease can be created using rotenone, a mitochondrial I-complex inhibitor, which induces dopaminergic neuronal death. Rats are therefore treated with DHA to test its effectiveness, followed by rotenone. Pretreating these rats with DHA protects dopaminergic neurons against this mode of death [133].

In an experimental rat model of Parkinson’s disease created with 1-methyl-4-phenyl-1,2,3,6-tetrahydropyridine (MPTP), DHA induces phosphorylation of the Akt protein, thus activating the Akt-dependent survival pathway, and also acts on the Bcl-2 pathway [134]. The same team also showed that DHA would decrease lipid oxidation in the brain [135].

In a model using the herbicide paraquat that induces dopaminergic neuron loss through the excessive production of ROS, DHA inhibits neuronal cell death by increasing glutathione homeostasis via Nrf2 [136].

Plasmalogens, glycerophospholipids containing vinyl ether linkage at the sn-1 position, can attenuate the neuronal cell death in Neuro-2A cells upon the serum starvation. The plasmalogens would activate the PI3K/Akt-signaling pathway by promoting its phosphorylation and the MAPK/ERK 1/2 pathway. Plasmalogens are also able to inhibit nutrient-deprivation-induced neuronal cell death in the hippocampus via inhibition of caspase-9 and caspase-3 activity [137].

Mice treated with MPTP and fed a diet containing 0.8% ethyl EPA (% of total diet weight) showed a reduction in striatal TNF-α and IFN-γ proteins [138]. The IL-10 protein is reduced in the midbrain following treatment with ethyl EPA. On the other hand, the expression of COX-2 and calcium-dependent cytosolic PLA2, enzymes involved in inflammatory signaling, are not altered.

## 7. Conclusions

**Figure 2:**
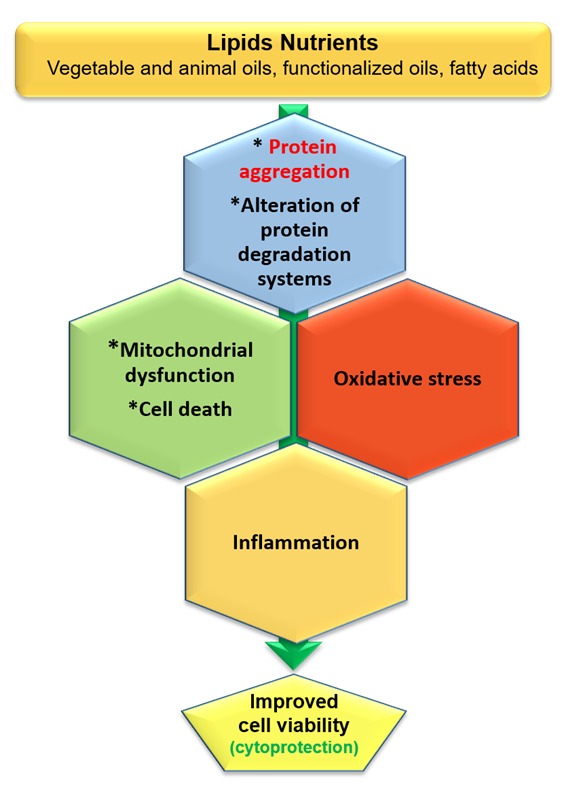
The potential of lipid nutrients to prevent Parkinson’s and Alzheimer’s disease. Several lipid nutrients, including vegetable and animal oils, functionalized oils and fatty acids, could be used to treat or attenuate Parkinson’s and Alzheimer’s disease. These disorders have in common protein aggregation and the alteration of protein degradation systems, which can trigger mitochondrial dysfunction and oxidative stress, leading to cell death and inflammation. There are now several in vitro and in vivo arguments that lipid nutrients can be efficient in the prevention of neurodegeneration associated with protein aggregation (β-amyloids in Alzheimer’s disease and α-synuclein in Parkinson’s disease). In the future, the cytoprotective activity of lipid nutrients could be enhanced by micro- or nanoencapsulation. Nanotherapy targeting the mitochondria may be considered (Targeted Organelle Nanotherapy (TORN therapy)).

Parkinson’s and Alzheimer’s disease have common physiopathological processes, such as protein aggregation and accumulation and altered protein breakdown systems, mitochondrial malfunction associated with cell death and oxidative stress. To prevent these side effects, some nutrients could be used as nutraceuticals or a as part of functionalized foods. There are indeed several lines of evidence that oils and fatty acids used alone could be employed because of their actions on protein aggregation, dysfunctional protein degradation systems, cell death, oxidative stress and/or inflammation to prevent or slow down the development of these diseases (Figure 2). Furthermore, interactions between nutraceutical products could have inverse, negative results; it is important not to value the additive or synergistic effects of combination products in vivo without testing them in animal models and human clinical studies. In the future, micro- and nanoencapsulation of oils and fatty acids associated with targeted therapies could help to optimize the efficiency of these compounds, whose activities depend both on the degree of degradation in the gastrointestinal tract and on the passage through the blood-brain barrier. Lipid nutrients are certainly a valuable source for developing therapies, but there are also other molecules such as polyphenols, whose properties have already been demonstrated in neurodegenerative diseases and which could be used in combination with these lipids [139,140].

## Abbreviations

^.^OH	hydroxyl radicals
6-OHDA	6-hydroxydopamine
7KC	7-ketocholesterol
AA	arachidonic acid
ALP	autophagy-lysosome pathway
APP	Amyloid precursor protein
Aβ	amyloid beta
BACE	beta-site APP cleaving enzyme
CHO	Chinese hamster ovary cells
CMA	chaperone mediated autophagy
CNS	central nervous system
DHA	docosahexaenoic acid
DHA-PC	DHA-enriched phosphatidylcholine
DHA-PS	DHA-phosphatidylserine
EPA	eicosapentaenoic acid
EPA-PC	EPA-enriched phosphatidylcholine
GSH	reduced glutathion
GSH-PX	glutathion peroxydase
H_2_O_2_	hydrogen peroxide
HFD	high-fat diet
hiPSC	human-induced pluripotent stem cell
IL	Interleukin
LA	linoleic acid
LRRK2	Leucine-rich repeat kinase 2
MCP1	monocyte chemoattractant protein-1
MHCII	major histocompatibility complex type II
MIP-1α	macrophage inflammatory protein-1α
MPP(+))	1-methyl-4-phenylpyridinium
MPTP	1-methyl-4-phenyl-1,2,3,6-tetrahydropyridine
NMDA	N-methyl-D-aspartate
NO.	nitrogen monoxide
O_2_^.−^	superoxide
OA	oleic acid
PET	Positron emission tomography
PINK1	PTEN-induced putative kinase 1
PUFA	polyunsaturated fatty acids
RONS	reactive oxygen nitrogen species
ROS	reactive oxygen species
SAP	saporin
SOD	superoxide dismutase
T-AOC	total antioxidant capacity
TNF-α	tumor necrosis factor-α
TOM	translocase of the outer membrane
Tregs	regulatory T-lymphocytes
UPS	ubiquitin-proteasome system
α-LNA	α-linolenic acid
γ-LNA	γ-linolenic acid
TORN	Targeted Organelle Nanotherapy
